# Short winters threaten temperate fish populations

**DOI:** 10.1038/ncomms8724

**Published:** 2015-07-15

**Authors:** Troy M. Farmer, Elizabeth A. Marschall, Konrad Dabrowski, Stuart A. Ludsin

**Affiliations:** 1 Department of Evolution, Ecology and Organismal Biology, Aquatic Ecology Laboratory, The Ohio State University, 230 Research Center, 1314 Kinnear Road, Columbus, Ohio 43212, USA.; 2 School of Environment and Natural Resources, The Ohio State University, 473D Kottman Hall, 2021 Coffey Road, Columbus, Ohio 43210, USA.

## Abstract

Although climate warming is expected to benefit temperate ectotherms by lengthening the summer growing season, declines in reproductive success following short, warm winters may counter such positive effects. Here we present long-term (1973–2010) field patterns for Lake Erie yellow perch, *Perca flavescens*, which show that failed annual recruitment events followed short, warm winters. Subsequent laboratory experimentation and field investigations revealed how reduced reproductive success following short, warm winters underlie these observed field patterns. Following short winters, females spawn at warmer temperatures and produce smaller eggs that both hatch at lower rates and produce smaller larvae than females exposed to long winters. Our research suggests that continued climate warming can lead to unanticipated, negative effects on temperate fish populations.

## Abstract (web summary)

Reductions in winter duration under climate change may have negative consequences for ectotherms adapted to seasonal temperature regimes. Here Farmer *et al*. show how short winters have, in part, caused failed lake fishery recruitment by altering spawning phenology and lowering the quality of eggs and larvae.

## Graphical abstract

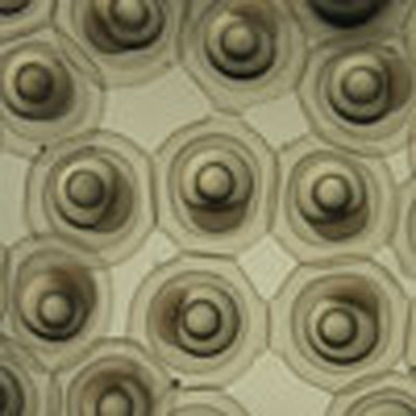

Climate change is most evident in northern temperate and arctic ecosystems, where it is prolonging historically short growing seasons^1^,^2^. Longer spring-through-fall growing seasons in northern ecosystems are predicted to have positive effects on temperate ectotherms by increasing fitness^3^ through a number of pathways, including increased positive growth^3^,^4^ and reduced overwinter mortality^5^. However, the corresponding reduction in winter duration may counter these positive benefits by presenting a mismatch between altered seasonal thermal regimes and the highly evolved seasonal physiology and life history of temperate ectotherms^6^,^7^.

For ectotherms, seasonally specific thermal regimes and photoperiod are important for successful reproduction^1^,^6^. For example, in many temperate fishes, patterns in temperature and photoperiod serve as cues for the timing of reproductive events (for example, ovulation and spawning)^7^. A shortened winter (and early spring onset) may alter the phenology (that is, timing) of spawning, eventual larval fish emergence and prey production, thereby increasing the potential for a mismatch between first-feeding larvae and their planktonic prey during spring^8^,^9^,^10^, reducing survival of larvae to the juvenile stage. In addition, temperature can be a critical determinant of proper reproductive development (for example, vitellogenesis) in temperate fishes^7^. Experimental and field-observational research in North American and European ecosystems has documented reductions in fecundity and egg quality when adult females do not experience a prolonged period of cold temperatures during the winter before spawning^11^,^12^,^13^,^14^. Because the number of viable eggs produced annually sets the absolute maximum potential cohort size for that year^15^, those aspects of climate change that affect fecundity and egg quality may strongly impact population dynamics and viability^16^,^17^. Climate change, therefore, may adversely affect reproductive success of temperate fishes by creating seasonal thermal regimes that are sub-optimal for reproductive development and by decoupling evolutionarily predictable relationships between temperature and photoperiod during seasonal transitions^6^, which largely govern the timing of reproduction.

A fuller understanding of how continued climate change, including the increase in frequency of short, warm winters in temperate regions^1^,^2^, will influence fish recruitment and population dynamics via reproductive pathways is essential to fisheries management and conservation efforts^16^. This need is especially evident given the focal species and experimental design used by previous studies that have investigated linkages between climate warming and fish reproductive success^11^. For example, 46% of the 41 reviewed studies^11^ that found climate change negatively affected fish reproductive success involved salmon species, which have life-history attributes that differ from most temperate fishes. Further, because 83% of these studies did not examine consequences beyond hatching^11^, the potential for these negative impacts during the egg stage to carry over to future life stages or to have population-level impacts remains largely unexplored. The capacity for winter warming to drive the dynamics of wild, temperate fish populations through effects on reproductive success also remains unknown because most (88%) of previous studies used domesticated or lab-reared broodstock (not wild fish) and many used unnatural photothermal conditions^11^.

Here we report findings from field studies and a laboratory experiment, which support our hypothesis that climate change will negatively affect reproductive success of temperate fishes by increasing the frequency of short, warm winters. Our investigation focused on yellow perch, which (i) develops ovaries during winter and spawn during spring, a common reproductive strategy in temperate regions^7^ and (ii) is ecologically, economically and culturally important across North America, including in our study system, Lake Erie. We first show that recruitment to the juvenile (age-0) stage, which is a strong predictor of both recruitment to the fishery at age-2 and future lifetime fishery harvest of that cohort, consistently failed following short, warm winters during 1973–2010. We then use experiments and supplemental field collections to show that, following short, warm winters, female yellow perch spawn at warmer temperatures and produce smaller eggs that both hatch at lower rates and produce smaller larvae than females exposed to long, cold winters. In addition to providing an explanation for the observed pattern of low recruitment to Lake Erie's yellow perch fisheries following short, warm winters, our work identifies several unanticipated reproductive pathways by which climate change may negatively affect temperate fish populations.

## Results

### Low juvenile abundance follows short winters

**Figure 1:**
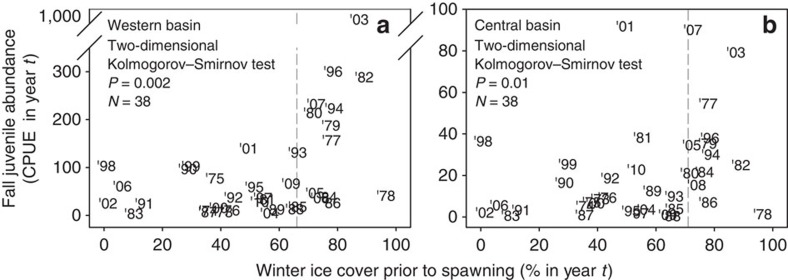
Lake Erie juvenile yellow perch abundance versus mean winter ice cover, 1973–2010. Winter ice cover was measured as the per cent of lake surface area covered by ice^46^ during the winter (February-March) before spawning in (**a**) western and (**b**) central Lake Erie in year *t* (year in which each cohort was hatched). Two-dimensional Kolmogorov–Smirnov tests^53^ determined whether changes in the variance of juvenile abundance were related to ice cover from the previous winter. This distribution-free test identifies a threshold value of ice cover (indicated by vertical dashed lines) that maximizes the difference in variance of juvenile abundance between the two sides of the threshold. *P* values indicate if the variance of juvenile abundance significantly differs between the two sides of the threshold. Above the threshold, juvenile abundance may be high or low, but below the threshold, only low abundances occur. Thus, winter duration appears to ‘set the stage' for future high recruitment to the fishery. Juvenile (age-0) abundance was determined from annual, fisheries-independent Ohio Department of Natural Resources-Division of Wildlife bottom trawling surveys conducted during October of year *t*, and is presented as catch-per-unit-effort (CPUE; # of individuals per trawl minute) for both (**a**) western and (**b**) central Lake Erie.

**Figure 2:**
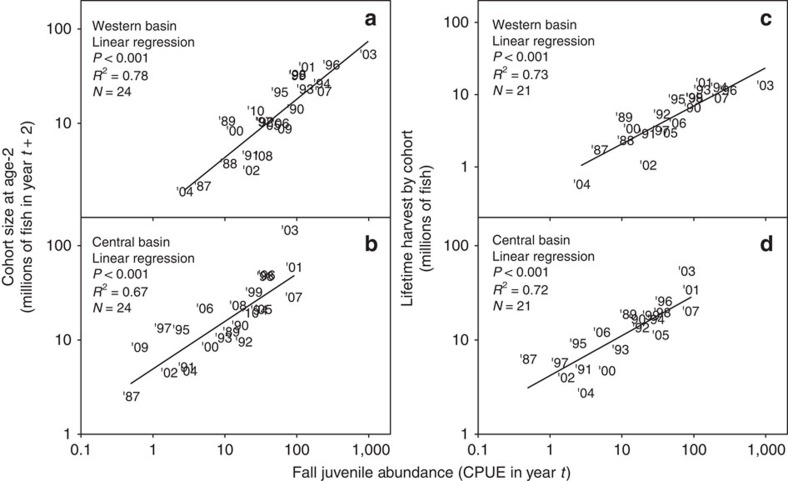
Lake Erie yellow perch cohort size at age-2 and lifetime harvest by cohort plotted against juvenile abundance, 1987–2010. Juvenile(age-0) abundance (1987–2010) was determined from annual, fishery-independent Ohio Department of Natural Resources-Division of Wildlife bottom trawling surveys conducted during October in (**a**) western and (**b**) central Lake Erie, and is presented as CPUE (# of individuals per trawl minute) in year *t* (year in which each cohort was hatched). Estimates of cohort size at age-2 in year *t*+2 for (**a**) western and (**b**) central Lake Erie (1987–2010) were generated by the Great Lakes Fishery Commission's Lake Erie Yellow Perch Task Group^48^. Lifetime harvest by cohort in (**c**) western and (**d**) central Lake Erie is the cumulative harvest by commercial fishers and recreational anglers (1987–2008) at ages 2–5 (that is, years *t* +2 through *t*+5)^48^. Linear regression was used to relate cohort size at age-2 and lifetime harvest by cohort to fall juvenile abundance. Results indicate fall juvenile abundance is a good proxy for future recruitment to age-2, when yellow perch typically become reproductively mature and enter the fishery. Thus, recruitment to the fishery is strongly influenced by factors operating prior to first fall of life, with strong recruitment events supporting Lake Erie's commercial and recreational fisheries for many years afterwards.

Retrospective analysis of long-term (1973–2010) fishery-independent data from Lake Erie revealed a consistent pattern of low juvenile abundance following short, warm winters. In both western and central Lake Erie, we detected a positive threshold relationship between an index of winter longevity (February to March ice cover, which is highly correlated with mean winter air temperature; Pearson correlation: *r*=−0.86, *P*<0.001, *N*=38) and the abundance of juvenile yellow perch in the subsequent fall (Fig. 1). Short, warm winters with little ice cover were consistently followed by weak recruitment to the juvenile (age-0) stage. By contrast, high juvenile abundances occurred only after long, cold winters. As yellow perch juvenile abundance in Lake Erie is a strong predictor of both recruitment to the fishery at age-2 and future lifetime fishery harvest of that cohort (Fig. 2), low juvenile abundances following short, warm winters translate into low adult abundances and reduced fishery harvests in future years.

### Short winters reduce egg and larval quality

**Figure 3:**
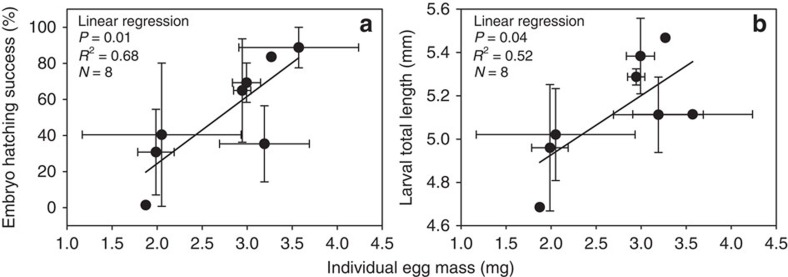
Lake Erie yellow perch (a) embryo hatching success and (b) larval size-at-hatching versus individual egg mass when exposed to a short and long winter in the laboratory. Winter-duration treatments used in our experiment were based on historical (1994–2010) field measurements of water temperature from a central Lake Erie water intake located near Cleveland, OH, USA (41° 32′ 53′′ N, 81° 44′ 60′′ W). The short winter duration (52 days) was similar to the number of days below 5 °C recorded during winter 1999 (*N=*59 days below 5 °C), which was, until 2012, the warmest winter on record for Ohio (1895–2013)^56^. Our long winter duration (107 days) was equal to the mean number of days below 5 °C observed during 1994–2010. Each winter duration treatment had six replicate tanks, although fertilized samples for embryo hatching success and larval size-at-hatching were only obtained from four replicate tanks in each treatment. All data are presented as tank means±1 s.e. (1–3 observations per tank). Large eggs hatched at higher rates (linear regression: embryo hatching success=0.37·egg mass−0.5) and produced larger larvae (linear regression: larval total length=0.27·egg mass+4.3) than small eggs.

Laboratory experimentation revealed that females exposed to a long winter produced larger eggs than those exposed to a short winter (one-way ANOVA: F_1,7_=6.1, *R*^2^=0.50, *P*=0.048, *N=*8, Levene's test for homogeneity of variance *P*=0.27). Further, relative to small eggs, large eggs had a higher energetic (linear regression: *R*^2^=0.90, *P*<0.001, *N=8*) and total lipid (linear regression: *R*^2^=0.63, *P*=0.03, *N=*8) content, and hatched at higher rates (Fig. 3a) and produced larger larvae (Fig. 3b). Female spawner traits (size, age and energetic condition) did not influence egg quality (that is, egg size, egg energetic and lipid content), embryo hatching success or larval size-at-hatching (Supplementary Table 1). These egg and larval responses also were unrelated to incubation temperatures (Supplementary Table 1), which were maintained at optimal levels during the experiment, pointing to winter-duration effects on gametogenesis as the underlying driver of observed differences between experimental treatments. Fecundity was driven primarily by female size (Supplementary Table 1) and did not differ between long and short winter-duration treatments (one-way ANOVA: *P*=0.44, *N=*8). All females lost somatic mass during the experiment, with this loss not differing between long and short winter-duration treatments (one-way ANOVA: *P*=0.62, *N=*8).

### Short winters disrupt spawning phenology

**Figure 4:**
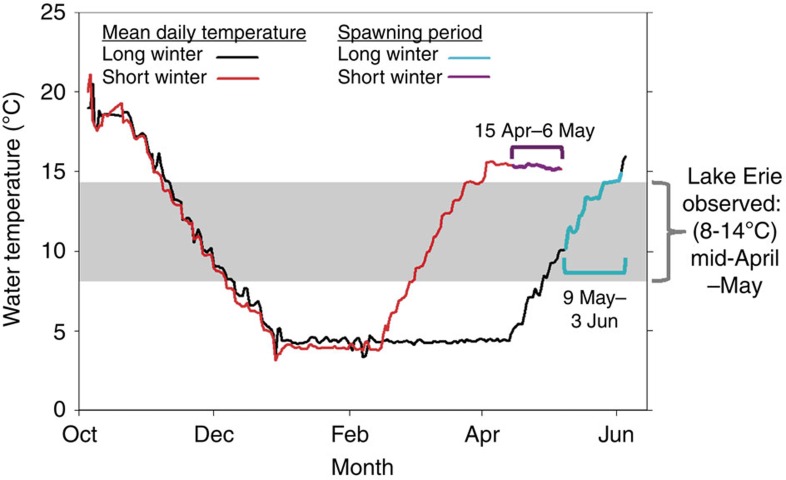
Mean daily water temperature from short and long winter-duration treatments used in a controlled laboratory experiment with Lake Erie yellow perch. Winter-duration treatments used in our experiment were based on historical (1994–2010) field measurements of water temperature from a central Lake Erie water intake located near Cleveland, OH, USA (41° 32′ 53′′ N, 81° 44′ 60′′ W). The short winter duration (52 days) was similar to the number of days below 5 °C recorded during winter 1999 (*N=*59 d below 5 °C), which was, until 2012, the warmest winter on record for Ohio (1895–2013)^56^. Our long winter duration (107 days) was equal to the mean number of days below 5 °C observed during 1994–2010. The duration of spawning is shown for each treatment. Labels indicate the dates and water temperatures during which females spawned. Despite earlier arrival of suitable spawning temperatures (8–14 °C within grey shading^18^; this study) following a short winter, females did not initiate spawning at these ‘normal' temperatures in our experiment, and instead spawned at warmer temperatures during the ‘normal' spawning period (that is, mid-April through May^14^,^18^).

**Figure 5:**
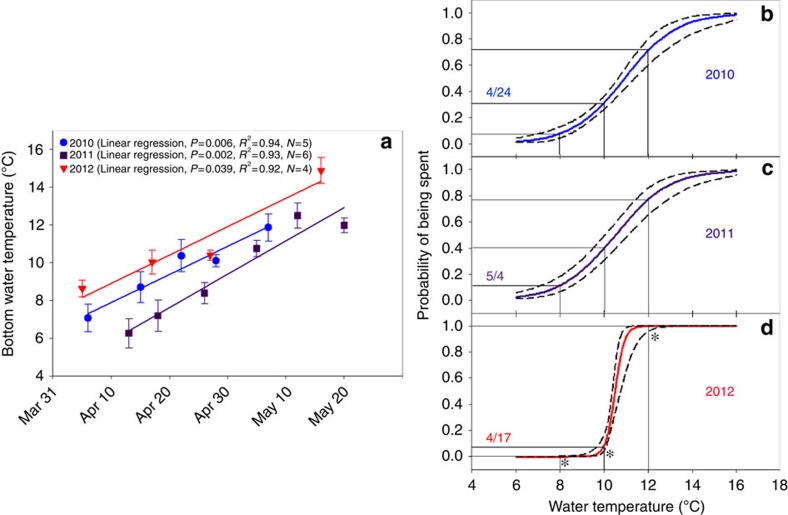
Relationship between (a) bottom water temperature and date and (b–d) the probability that a yellow perch female was spent and bottom water temperature during spring 2010–2012 in western Lake Erie. Bottom water temperatures in (**a**) are presented as means±s.d. for each sampling event (2010: *N*=9–20, 2011: *N*=9–20, 2012: *N*=4–9 observations per sampling event) and related to date, in each year, with linear regression. Coloured lines in (**b**–**d**) were determined by logistic regression; dashed lines in **b** indicate 95% confidence intervals. Dates in (**b**–**d**) identify the date on which bottom water temperatures reached 10 °C in each year (determined from regressions conducted on data in (**a**)). Solid black lines in (**b**–**d**) indicate probabilities determined for 8, 10 and 12 °C each year. During (**b**) 2010 and (**c**) 2011, spawning had begun by the time the temperature reached 8 °C and was well under way by the time temperatures reached 10 °C. During (**d**) 2012, the year with the earliest spring, fish were not yet spawning when the temperature reached 8 °C, and were barely spawning at 10 °C. Instead, spawning was concentrated at warmer temperatures than during (**a**) 2010 and (**b**) 2011. The asterisks indicate that the probability of females being spent at 8, 10 and 12 °C differed in 2012 (*P*<0.05; 95% confidence intervals do not overlap) when compared with 2010 and 2011, which had similar probabilities across all temperatures (*P*>0.05; 95% confidence interval overlap).

Beyond finding that short winters reduced egg size, egg energetic and lipid content, embryo hatching success and larval size-at-hatching, we documented differences in spawning phenology in response to spring warming in both the laboratory and wild. In the short winter-duration treatment, females did not spawn at the temperatures commonly observed for this species in the wild (8–14 °C)^14^,^18^, which occurred during mid-March in the short winter-duration treatment of our experiment. Instead, these females delayed spawning until the historically more typical spawning-period (mid-April through May)^18^, even though temperatures (∼15 °C) exceeded those typically observed during yellow perch spawning in the wild (Fig. 4). A similar phenomenon was observed in Lake Erie. During 2010 and 2011, spawning had begun by the time temperatures reached 8 °C and was well under way by the time temperatures reached 10 °C (Fig. 5). During 2012, the year with the earliest spring, fish were not yet spawning when the temperature reached 8 °C, were barely spawning at 10 °C and instead concentrated their spawning at warmer temperatures than during 2010 and 2011 (Fig. 5).

## Discussion

By empirically demonstrating that the reproductive success of wild yellow perch declines following short, warm winters, we identify a novel process that can explain how climate warming has been, in part, driving low juvenile abundance and failed recruitment to Lake Erie's fisheries after warm winters. Most striking was the observed reduction in egg size, egg energetic and lipid content, and embryo hatching success following short, warm winters, with no corresponding effect on individual fecundity. While previous studies have documented reductions in fecundity^11^, spawning^14^ and egg viability^11^,^12^ in response to a variety of shortened winter thermal regimes, the majority of these studies used unnatural temperature and photoperiod (that is, photothermal) regimes^14^ and domesticated broodstock^11^. Our study is among the first to document reductions in egg size, egg energy and lipid content, embryo hatching success and larval quality in wild individuals in response to environmentally relevant winter photothermal regimes.

Across the Great Lakes, short-term variability in yellow perch recruitment events has been linked to multiple causal factors, including predator abundance^19^, zooplankton availability^20^, spring warming rate^21^ and temperature^22^. However, ability to forecast yellow perch recruitment over the long-term has remained elusive in Lake Erie, likely owing to a complexity of human-driven perturbations to the ecosystem that have occurred during the past several decades (for example, oligotrophication^23^ and dreissenid mussel invasions^24^, and their subsequent restructuring of the food web^25^). Our historical analyses, however, point to winter severity as being a consistent driver of yellow perch recruitment during this period of ecosystem change, with recruitment being negatively affected by short, warm winters. Further, our laboratory experiment and field investigation have revealed that short, warm winters likely have been controlling this long-term recruitment relationship by reducing egg size and hatching success, as well as by altering spawning phenology.

Whether short, warm winters led to reduced egg size, egg energetic and lipid content, and embryo hatching success through metabolic or maternal endocrine pathways requires further investigation. The difficulty in delineating between these mechanisms is in part due to both sets of processes being controlled by ambient temperature^26^,^27^. For example, for successful reproduction to occur, the metabolic demands of reproduction must be met^27^. In this way, one potential explanation for large eggs being produced after long winters is that the prolonged period of cold temperatures allows for more energy to be allocated to reproduction than under warmer conditions, when maintenance metabolic demands are elevated^27^,^28^,^29^. Most certainly, additional research into how seasonally specific metabolic rates^30^ and food availability during winter affect energy allocation by females to reproduction (sensu^29^) in temperate fishes would be worthwhile.

Alternatively, by disturbing the proper timing of hormone and sex steroid production^26^, warm temperatures during short winters may have disrupted vitellogenesis, thereby reducing maternal investment in oocytes^12^,^13^,^31^,^32^. Indeed, thermal inhibition of steroid hormone levels has been documented in a diversity of fishes and under a wide range of thermal conditions^11^,^31^, although species- and location-specific thermal thresholds ultimately appear to control the response of each species^31^. In salmonines, for example, elevated temperatures have been shown to cause conformational changes in proteins (for example, follicle-stimulating hormone, luteinizing hormone) and their enzyme receptors, as well as cause steroid hormones to form water-soluble conjugates that may not easily pass through cell membranes to reach their receptors or that are more easily removed from the plasma by kidney filtration^26^. For yellow perch and other temperate fishes that develop eggs during winter and spawn during spring, previous research also has clearly demonstrated that parental exposure to warm temperatures during vitellogenesis can reduce plasma concentrations of steroid hormones in females^32^. Such reductions, in turn, likely underlie observed reductions in the quantity and quality of oocytes produced by temperate fishes following parental exposure to elevated temperature^11^,^12^,^13^,^14^,^26^,^31^,^32^.

While the exact physiological mechanisms underlying failed Lake Erie yellow perch recruitment are unknown, our results, and those of previous studies^11^,^12^,^13^,^14^,^26^,^31^,^32^, clearly indicate that elevated temperatures during vitellogenesis, final oocyte maturation and ovulation can negatively affect egg quality, embryo hatching success and, ultimately, the total number of offspring produced. For this reason, we encourage future research aimed at determining the physiological mechanisms responsible for these declines in reproductive success, as well as the species-specific temperature thresholds at which such mechanisms operate.

In addition to reduced embryo hatching success (and hence, reduced larval production), females exposed to a short, warm winter produced smaller larvae than those exposed to a long, cold winter. This finding is important in Lake Erie because of the recent establishment of a highly abundant invasive predator (white perch, *Morone americana*), which has been estimated to consume large numbers of yellow perch larvae^33^, particularly slow-growing ones^34^. Similar observations of both a large size-at-hatching^35^ and fast larval growth^36^ conferring future growth and survival advantages abound in both the freshwater and marine literature.

Altered spawning phenology offers yet another pathway by which short, warm winters could weaken annual recruitment. In our study, female yellow perch that were exposed to a short winter (and hence, earlier arrival of spring temperatures) did not initiate spawning at their ‘normal' temperatures in either the laboratory or Lake Erie. Instead, following a short winter, females initiated spawning at warmer temperatures than normal, which occurred during the typical spawning period (mid-April through May).

This delay in spawning following a short winter could negatively affect temperate species such as yellow perch in two vastly different ways. The prolonged period of elevated water temperature during a ‘delayed' spawning season may reduce sperm quality (for example, motility). Indeed, low sperm quality was associated with a prolonged, warm spawning season in a congener of yellow perch (Eurasian perch *Perca fluviatilis*)^37^, as well as other temperate fish species (for example, brown trout *Salmo trutta*^38^). Because poor sperm quality also may have contributed to failed reproduction following short, warm winters in Lake Erie, further investigation into climate change effects on male gamete quality is warranted.

Inability to fully adjust spawning in response to early spring warming also would be expected to result in low larval survival, if hatching does not coincide with prey production^8^,^9^,^10^. When the timing of peak zooplankton production in temperate regions primarily tracks temperature (for example, ref. 39), winter warming would be expected to negatively affect larval survival and subsequent population growth potential^8^ in the absence of any rapid shift in spawning phenology. While latitudinal variation in spawning times has been documented for our study species in North America (for example, spawning occurs during late February to March in Maryland versus May into June in Canada^40^), which suggests that the evolutionary potential for yellow perch to adapt to climate warming exists, future research would be required to fully understand whether the rate of adaptation in Lake Erie yellow perch can keep pace with the rate of winter warming. Unfortunately, similar to yellow perch in our study, most temperate fishes do not appear to be adjusting their spawning phenology fast enough to keep pace with the rate of warming^41^.

Identifying the environmental cues that initiate and modulate spawning will be critical to efforts aimed at determining whether yellow perch (and species like it) can adapt rapidly enough to keep pace with continued climate warming. At present, temperature and photoperiod are widely considered the predominant environmental cues initiating the final stages of maturation and spawning in many temperate fishes^7^,^42^. While our study was not designed to determine the extent to which temperature or photoperiod acted as deterministic or modulating influences on spawning time, our laboratory and field results clearly show that accelerated temperature during spring was not, by itself, sufficient to initiate spawning in females following a very short winter (for example, 2012). Given recent physiological research, which has shown that steroid hormone production and ultimately reproductive processes in temperate fishes can be indirectly regulated by photoperiod through its effect on melatonin production (see review by ref. 42), we find it highly plausible that photoperiod is playing a role in cueing final maturation and spawning. However, because few studies have successfully isolated the effects of seasonal photoperiodic and thermal cues on the final stages of maturation and ovulation in temperate fishes^7^,^42^, we encourage further research in this arena.

Although the endocrine regulation of gametogenesis in teleosts is complex and species-specific^7^, our conclusions seem applicable to other ecologically and economically important spring-spawning temperate fishes with potentially similar photothermal requirements. For example, a recent classification of temperate fishes into functional groups based on their reproductive strategies grouped yellow perch with a wide diversity of early spring spawning species (for example, Eurasian perch, walleye *Sander vitreus*, pike-perch *Sander lucioperca*)^7^. These species also require a long, cold winter to complete vitellogenesis, followed by a rise in spring temperature, with photoperiod also appearing important to the completion of ovulation and spawning^7^. As winter temperatures in temperate and arctic regions have been warming faster than summer temperatures^1^,^2^, an urgent need exists to understand the extent to which our findings are applicable to other temperate fishes with similar reproductive strategies. Furthermore, given that our work has suggested pathways by which both short winter duration and early spring arrival may negatively affect reproductive success, we encourage future research that is specifically designed to test for the independent effects of these two correlated but independent, meteorological occurrences on temperate fish reproductive success.

Our findings are significant in that they show how shorter winters can negatively affect a group of fishes previously expected to benefit from climate warming. We do not dispute the many positive benefits that longer growing seasons can provide to temperate fishes^3^,^4^; however, if the total number of offspring has already been severely limited by reduced embryo hatching success and low larval survival following short, warm winters, the relative strength of these positive benefits may be functionally limited. In this manner, we expect continued winter warming, through its effects on reproductive success, to become a key driver of recruitment success in temperate ectotherms. To accurately predict how climate change will affect temperate fishes, we must understand how both longer growing seasons and shorter winters will affect these species' highly evolved, seasonally specific life histories. In assessing the effects of climate change on ectothermic animal populations, we suggest that future studies give considerably more attention to the potentially negative effects of winter warming on reproductive success.

## Methods

### Study system and species

As with many freshwater and marine systems globally, the Laurentian Great Lakes already have begun to warm^43^. Over Lake Erie, average winter air temperature (mean daily temperatures from Toledo, OH [NOAA-NCDC station #94830] and Cleveland, OH [NOAA-NCDC station #14820]) averaged across both sites and smoothed using a 3d moving average) during 1956–2012 increased by 2.4 °C (Pearson correlation coefficient: *r*=0.39, *P*=0.003, *N*=57) and the annual average number of days with air temperatures below freezing decreased by 16 days (*r*=−0.34, *P*=0.01, *N*=56). Average air temperatures also are expected to continue to rise in the Great Lakes basin, with predicted increases of 2–5 °C during winter by 2050, depending on emission scenarios^44^. Further, in at least one of the Great Lakes, water temperature has been increasing twice as fast as air temperature^45^, largely due to a reduction in winter ice cover, which has decreased 72% over the past four decades^46^.

Yellow perch is a common, coolwater iteroparous fish that is widespread across the Atlantic, Great Lakes and Mississippi River basins of North America. This species is particularly important in the Laurentian Great Lakes basin, where it serves as an important consumer in the middle of the food web^47^ and supports valuable commercial and recreational fisheries. Yellow perch supports Lake Erie's largest commercial fishery and second most valuable recreational fishery^48^. Recruitment to the fishery has been quite variable in Lake Erie over the past several decades^48^, a period of time during which variability in winter ice cover also has been increasing^49^.

In Lake Erie, as across much of its range, yellow perch develop ovaries during winter months^49^ and spawn during spring (mid-April through May^18^) across the lake, with different local spawning stocks that mix in the open lake as adults^50^. Embryos hatch and develop into pelagic larvae during May through June, depending on lake basin, with larvae feeding solely on zooplankton before becoming demersal omnivores after about 25–35 days of age^51^. Juveniles recruit to agency (fishery-independent) assessment gear by August of their first year of life and eventually become reproductively mature and enter the fishery at age-2. Juvenile abundance is a strong predictor of recruitment to the fishery at age-2. In turn, because strong recruitment events to the fishery at age-2 support the fishery for many years afterwards, factors such as winter duration, which influence juvenile abundance, leave a long legacy that is evident in fishery harvest.

### Historical analysis of recruitment in relation to ice cover

*Juvenile abundance indices*. Analysis of Lake Erie yellow perch population dynamics (1973–2010) used juvenile (age-0) catch rate data (number of juveniles caught per min of bottom trawling) generated annually during an October fishery-independent assessment survey conducted by the Ohio Department of Natural Resources-Ohio Division of Wildlife (ODNR-DOW), as coordinated by the Great Lakes Fishery Commission's Lake Erie Yellow Perch Task Group^48^. As sampling was conducted by multiple vessels during these years, we applied vessel-specific fishing power corrections to juvenile abundances from 1982 to 2010 to standardize catches^52^. Juvenile abundances from 1973 to 1981 had no fishing power corrections applied, due to a lack of studies comparing older and modern research vessels. However, sampling biases do not appear to underlie observed relationships (see abundance-ice cover analysis).

Because the ODNR-DOW survey design changed during the past 35+ years, we used yellow perch data only from 12 fixed, historical sites, sampled consistently during this time. Historical sites were spread across the Ohio waters of western (*N=*4) and central (*N=*8) Lake Erie^23^. Annual juvenile abundances from these historical sites were strongly correlated with overall annual abundances calculated using all sites in both western (Pearson correlation: *r*=0.92, *N*=24; ∼80 sites per year since 1987) and central Lake Erie (Pearson correlation: *r*=0.94, *N*=21; ∼40 sites per year since 1990), indicating that historical sites closely track population-level variation in juvenile yellow perch abundance.

*Ice cover*. We used ice cover trends from 1973 to 2010 for Lake Erie as a proxy for winter water temperature because no continuous data set of water temperature exists for our entire study period for either lake basin. Ice cover data were compiled from ice charts generated at approximately weekly intervals by the Canadian Ice Service and NOAA National Ice Center, which were subsequently summarized and interpolated into daily values of lake-area ice cover^46^. Because ice cover reaches its peak on Lake Erie during February through March^46^, we selected this timeframe to distinguish between years of low and high ice cover. Importantly, late winter (February-March) ice cover was highly correlated (Pearson correlation: *r*=−0.86, *P*<0.0001, *N*=37) with an independent index of mean winter air temperature (January–March; 1973–2010) developed for Lake Erie (see Study system and species section above), indicating that ice cover is a valid indicator of winter severity. This notion is further supported by analyses that related our basin-specific indices of yellow perch juvenile abundance to the most complete data sets (1969–1992) of daily water temperature for western (Put-it-Bay, OH) and central (Erie, PA) Lake Erie^43^. These analyses produced similar results to those obtained with our ice cover analyses in that we detected significant thresholds in mean winter water temperature below which recruitment could be either high or low, but above which, recruitment was always low (Supplementary Fig. 1).

*Abundance-ice cover analysis*. We used a two-dimensional Kolmogorov–Smirnov test^53^ to determine whether changes in the variance of juvenile abundance were related to our index of winter duration (that is, ice cover). *P* values from this distribution-free test indicate whether the variance of the dependent variable (that is, juvenile abundance) significantly differs between the two sides of threshold values in the independent variable (that is, ice cover). This analysis, spanning 1973–2010, included data collected from older research vessels (1973–1981) for which no fishing power corrections could be applied. Therefore, we repeated this analysis using only data from 1982 to 2010. Using only these recent data, with fishing power corrections applied, we obtained significant two-dimensional thresholds^53^ similar to those found using 1973–2010 data. Thus, we feel confident that sampling biases do not underlie our observed relationships.

*Stock size*. While yellow perch stock size (that is, number of age-3 and older mature yellow perch) varied markedly in both Lake Erie basins during 1973–2010, we do not feel that it is responsible for observed recruitment variation. In support of this notion, previous research showed that stock size explained <1% of the variation in yellow perch recruitment in both the western and central basins during 1973–2010 (ref. 54). Further, yellow perch spawning stock size generally declined in the west basin during this time period, whereas it increased in the central basin during this same period^48^,^54^. These opposing trends in stock size between adjacent lake basins also suggest that stock size was relatively unimportant to driving recruitment variation, given that we documented near identical responses of recruitment to variation in winter thermal regime.

### Timing of spawning in Lake Erie

*Spawner classifications*. To determine the timing of spawning in the wild, we sampled yellow perch weekly in central Lake Erie during spring (April–May) 2010–2012. Individuals were collected near Sandusky, OH (41° 30' N, 82° 37' W) by bottom trawling two nearshore-to-offshore transects. Transects were divided into five 1.5-m depth contours (5, 7.5, 9, 10.5 and >12 m) with two trawls conducted per depth contour. All female yellow perch collected during 2010 (*N=*551), 2011 (*N=*429) and 2012 (*N=*279) were euthanized, dissected and classified as either immature, mature (gravid but not spawning), spawning or spent, based on macroscopic inspection of gonads^55^: (1) immature females had a small thread-like transparent ovary; (2) mature females had clearly visible eggs with the ovary filling about two-thirds of the body cavity; (3) spawning females expressed egg ribbons with gentle pressure; and (4) spent females had empty, flaccid ovaries that were reddish grey.

*Spring temperatures*. While our study spanned only 3 years, these years varied greatly in spring (March–May) temperatures. Air temperatures varied greatly during the spawning period, with 2012 being the warmest year on record for OH (as well as for the contiguous United States). By contrast, 2011 and 2010 were ranked as the 92nd and 115th warmest spring on record, out of 119 annual observations (1895–2013)^56^. These differences were generally reflected in terms of water temperatures in Lake Erie during spring, which was measured before each trawl during 2011–2012 and for each depth contour during 2010. While spring water warming rates during 2010–2012 were similar among the 3 years (ANCOVA: *P*=0.67, *N=*15), spring water temperatures during 2010 and 2012 were warmer than 2011 (one-way ANOVA, Tukey's HSD *post hoc* test: 2010 versus 2011, *P*=0.01, *N=*11; 2012 versus 2011, *P*<0.001, *N=*10).

*Probability of spawning*. We used logistic regression to relate the number of mature females (that is, females in classification criteria 2–4 per above) to water temperatures. By including only mature females in our analyses, we were able to quantify how the transition of females from a gravid (that is, egg bearing) to spent condition varied in relation to spring water temperature. We created a binomial variable (0=gravid; 1=spawning or spent) to indicate the spawning status of each individual and used logistic regression (PROC GENMOD, SAS v. 9.3) to identify significant relationships within years (Supplementary Table 2). Confidence intervals (95%) generated for 8, 10 and 12 °C were used to determine whether the timing of spawning in each year differed in response to annual variation in temperature.

### Laboratory experiment

We conducted a controlled laboratory experiment with wild yellow perch to quantify the effects of winter duration on the timing of reproduction, fecundity, egg quality (that is, egg size, energetic and lipid content), embryo hatching success and larval-size-at-hatching. Below, we provide details regarding the fish used in the experiment, the experimental design and the treatment of the data.

*Collection and rearing of wild fish*. The central basin yellow perch population is the largest in Lake Erie, representing 66 and 70% of the population in terms of lake-wide abundance and biomass during 1987–2010 (ref. 48). For this reason, we collected male and female yellow perch for our experiment from central Lake Erie near Fairport Harbor, Ohio, USA (41° 46′ N, 81° 21′ W) during April–May 2011 (when sex could be determined by external examination). We collected all individuals via bottom trawling conducted aboard the ODNR-DOW's RV *Grandon*. Immediately upon collection, we placed all individuals into live wells and ‘fizzed'^57^ each fish with a hypodermic needle to prevent overinflation of the gas bladder^57^, a common phenomenon that can cause mortality in fish rapidly brought to the surface from depth. Fizzing reduced post-capture mortality and did not adversely affect long-term survival, as previously reported^57^.

After collection, all individuals were transported to the Aquatic Ecology Laboratory (AEL) at The Ohio State University (Columbus, OH, USA), where they were held until October 2011 in 2,500 l circular tanks in the AEL's outdoor pool facility, which provided constant aeration and flow-through, dechlorinated city water. After 2 weeks of acclimation at the AEL, all individuals were injected with a unique passive integrated transponder (PIT) tag (Biomark, Boise, ID, USA), which allowed us to monitor individual growth and maturation. We fed all individuals live fathead minnows (*Pimephales promelas*) during the pre-experiment holding period (April/May–October 2011). Water temperatures (measured daily with a YSI 550a, YSI Incorporated, Yellow Springs, OH, USA) varied from 7 to 8 °C during April, when the first yellow perch arrived in the facility, to a maximum of 23 °C during July, before falling to 18 °C by the first week of October. During this time, we made no effort to control water temperature so that it could closely mimic the seasonal variation observed in Lake Erie^43^. Water quality parameters (that is, ammonia, nitrite, nitrate and pH) were measured weekly and levels were always within acceptable ranges (that is, unionized ammonia<0.1 p.p.m.; nitrates<40 p.p.m.) during the pre-experiment holding period.

*Experimental design*. Our experiment was designed to quantify the effects of winter duration (number of days below 5 °C, treatment levels=52 and 107 days) on the timing of reproduction, fecundity, egg quality (that is, egg size, energetic and lipid content), embryo hatching success and larval size-at-hatching. Winter-duration treatments and the rates of fall cooling and spring warming (both 0.25 °C day^−1^) used in our experiment were based on historical (1994–2010) field measurements of water temperature from a central Lake Erie water intake located near Cleveland, OH, USA (41° 32′ 53′′ N, 81° 44′ 60′′ W). Our two winter-duration treatment levels were intended to simulate historical (107 days) and future (52 days) conditions for Lake Erie. Our short winter duration (52 days) was similar to number of days below 5 °C recorded during winter 1999 (*N=*59 days below 5 °C), which was, until 2012, the warmest winter on record for Ohio (1895–2013)^56^. Our long winter duration (107 days) was equal to the mean number of days below 5 °C observed during 1994–2010. After our simulated short winter, we halted spring warming when temperatures reached 15 °C because 1) yellow perch spawning in Lake Erie is typically completed by the time temperatures exceed 15 °C (ref. 18) and (2) previous laboratory work has documented reduced egg viability at temperatures>15 °C (ref. 58). As our objective was to assess the effects of winter duration on reproductive success, we view this approach as conservative. Continued warming above 15 °C would have likely caused reduced egg viability due to elevated spawning temperatures^58^.

Our experiment was conducted during October 2011 through June 2012 in two walk-in environmental-control chambers (located at the AEL) in which all physico-chemical conditions were controlled. Each chamber represented a single winter-duration treatment and contained a recirculating system with six 189 l tanks. Each recirculating system was supplied with continuous aeration along with physical and biological filtration to maintain water quality. Ammonia, nitrite, nitrate and pH were measured daily and water changes were conducted as needed to maintain high levels of water quality (that is, unionized ammonia<0.1 p.p.m.; nitrates<40 p.p.m.). Hobo loggers (Onset, Bourne, MA, USA) recorded water temperature (nearest 0.1 °C) every 2 h in each recirculating system, and temperature and dissolved oxygen were measured daily with a YSI 550a handheld meter (YSI Incorporated, Yellow Springs, OH, USA). Lighting in all rooms was provided by incandescent lights and controlled by a digital system that simulated daily reductions (during the fall) and increases (during winter and spring) in photoperiod so as to mimic the photoperiod at Cleveland, OH, USA (located at the south shore of the central basin). In addition, at dawn and dusk, 1 h of increasing or decreasing light intensity preceded day and night periods.

During the first week of October 2011, all fish were removed from outdoor holding tanks, briefly anaesthetised in buffered MS-222, measured (nearest 1-mm total length, TL), weighed (nearest 1-g wet mass) and scanned for PIT tags. A subset of nine females was euthanized with an overdose of MS-222, dissected and gonads weighed to quantify reproductive development at the initiation of our experiment. We assigned all remaining individuals to a winter-duration treatment and tank, using a random number generator. Each individual tank contained 12–15 individuals (8–9 females and 3–7 males; numbers varied to standardize initial tank biomass), for a total of 49 females and 28 males and 49 females and 26 males in the long and short winter-duration treatments, respectively. During the experiment, we fed all yellow perch daily maintenance rations of live fathead minnows. We determined maintenance rations from an existing bioenergetics model^59^, based on daily temperatures and the mass of individuals in each tank.

During the experiment, some females died (short winter *N=*19; long winter *N=*15). The majority (56%) of mortalities occurred during the first 3 weeks of October 2011 at the start of the experiment, likely due to transfer stress and failure to acclimate to indoor laboratory conditions. No mortalities occurred once spawning began in the spring (April–June 2012). Once spawning began, we euthanized another group of randomly selected female yellow perch in the short (*N=*14) and long (*N=*14) winter-duration treatments to assess reproductive development (results not presented). The remaining females in both the short and long winter-duration treatments all spawned, with some being hand-stripped (short winter *N=*7; long winter *N=*10) and some spawning in tanks (short winter *N=*9; long winter *N=*10). Thus, our sample sizes for fecundity, egg quality, embryo hatching success and larval-size-at-hatching for each treatment were based on the number of yellow perch that were able to be successfully hand-stripped of eggs.

To simulate the end of winter and onset of spring, we increased water temperatures based on historical Lake Erie water temperature data (see above). For our short winter-duration treatment, spring warming began at the end of February, whereas in our long winter-duration treatment, spring warming began during mid-April. During the spring warming period, we monitored females during the day and hand-stripped females of eggs once signs of ovulation were present. Although six tanks (that is, replicates) were used in each winter-duration treatment, we were able to hand-strip females from only four of the tanks in each treatment.

*Reproduction metrics*. We used a variety of metrics to assess the impact of winter duration on maternal investment in reproduction, considering both the number of potential offspring produced (that is, fecundity) and investment per offspring (that is, egg size, energetic and lipid content). We fertilized eggs using the dry method^60^ with composite milt samples from males in the same treatment (see Spawning and fertilization section). We also collected unfertilized egg samples to quantify fecundity, egg size and energetic and lipid content (see Fecundity and Egg quality sections). Fecundity gave us a measure of overall reproductive output, whereas egg size and energetic and lipid content served as measures of egg quality, as these metrics have been shown to be positively related to responses such as embryo hatching success^61^.

To assess the impact of egg quality on hatching success and larval quality, we incubated fertilized eggs and hatched them under controlled conditions. Embryo hatching success was calculated as the number of hatched larvae per number of fertilized eggs per sample (see Embryo hatching success section). All hatched larvae were preserved (3% buffered glutaraldehyde) for counts and measurements of larval size (see Larval size-at-hatching section).

Blinding of treatments from investigators during the experiment was not possible due to logistical constraints (that is, we were required to enter walk-in environmental chambers to conduct the experiment and differences in temperature between treatments were obvious). However, by labelling egg and larval samples with non-descript letter and number combinations upon collection, we were able to remain blinded to treatment assignments during the processing of samples for fecundity, egg quality, embryo hatching success and larval-size-at-hatching analyses.

*Spawning and fertilization*. To identify spawning activity as water temperatures increased at the conclusion of each winter-duration treatment, we monitored females every 30 min from first light until 3 h after last light for external signs of ovulation. Once females showed signs of ovulation (that is, swollen and slightly reddish genital papilla, bulging of the ovary towards the exterior), they were removed from tanks, briefly anaesthetised in MS-222, scanned for PIT tags, and dried with a cloth before applying gentle pressure to the abdomen to strip ovulated egg ribbons. Eggs were expressed into a dry pan and their mass recorded (nearest 0.1 g).

Subsamples from each individual's egg mass were fertilized, with a third subsample used to determine fecundity and quantify egg quality (that is, egg size, energetic and lipid content). For each fertilization event, two 2 g egg masses were fertilized using the dry method^60^ with a ‘fresh' composite of milt (that is, collected within 10 min of stripping eggs from each female) from three haphazardly selected males within the same winter-duration treatment as the stripped female. In this way, eggs from each female were fertilized with a new, unique composite milt sample. Milt from the three males was composited into Moore's extender^62^, where it was diluted 20-fold. Each 2 g egg mass was subsequently fertilized with a concentration of 100,000 spermatozoa per egg. We also analysed both individual and composite milt samples from each fertilization event for per cent sperm motility, duration of sperm motility and sperm density^62^. Results showed that none of these milt quality metrics were related to embryo hatching success (Pearson correlation: all *P*>0.05)^54^.

Some females released egg ribbons spontaneously in tanks, between our monitoring activities. We did not use these spontaneously released eggs to assess fertilization or embryo hatching success, as in-tank egg ribbon release can result in highly variable male fertilization success rates (that is, 40–85%)^63^. However, the timing of these spontaneous spawning events was recorded and used in our determination of spawning time.

*Timing of spawning*. The date and water temperature at which each female spawned was recorded, allowing us to determine spawning time differences between treatments. Following hand-stripping of eggs, each female was euthanized in an overdose of buffered MS-222, measured for TL and wet mass, and dissected to remove the spent ovary, which was subsequently weighed (nearest 0.1 g) for determination of somatic growth during the experiment (see Over-winter growth section). The sagittal otoliths of each female also were removed for age determination. Otoliths were cracked and sanded to the origin and briefly burned with an alcohol burner to aid in distinguishing annuli. Annuli were counted by three independent readers to determine age, following established guidelines^64^.

*Embryo hatching success*. To measure embryo hatching success, we first placed fertilized eggs into mesh-covered jars in upwelling California-style tray incubators. The incubators were supplied with water from a partially recirculating system equipped with a chiller to maintain water temperatures at optimal levels for yellow perch egg incubation (10–18 °C (ref. 65); see Supplementary Table 3) during the course of the experiment. Temperature and dissolved oxygen were recorded daily and eggs were monitored carefully for the first presence of eyed embryos. When fertilized eggs reached the eyed-embryo stage (8–10 days, depending on water temperature), the samples were moved into clear, plastic 500 ml jars that were filled with water and sealed to quantify embryo hatching success. An air stone in each jar provided vigorous aeration to assist with hatching. Hatching jars also were held in a bath of flow-through water to maintain stable temperatures. Incubation temperature for each egg mass was calculated as the mean daily temperature measured during incubation in both California-style tray incubators and plastic hatching jars.

Eggs were checked every 12–24 h for hatching. Once hatched larvae were visible, a 1,800 μm sieve was used to separate hatched larvae from un-hatched eggs. All hatched larvae were immediately euthanized and preserved in 3% buffered glutaraldehyde. After collection of hatched larvae, all unhatched eggs were returned to the hatching jar, with fresh water. The embryo hatching success of each fertilized egg sample was determined by dividing the total number of hatched larvae collected by the total number of eggs in each sample (determined following methods for fecundity estimation described below).

*Fecundity*. Fecundity was determined from egg ribbons of females that were hand-stripped and measured as the number of eggs per gram of ribbon. In brief, three subsamples of each egg ribbon (∼0.5 g each) were collected and weighed, and the number of eggs in each subsample was counted under a dissecting microscope. The total number of eggs produced by each female was estimated by multiplying the number of eggs per gram of ribbon by the overall ribbon mass^18^. While fecundity does not necessarily indicate fertility, which was tested with hatching tests, it can provide an objective measure of reproductive output.

*Egg quality*. In addition to using embryo hatching success as a measure of egg quality, we quantified egg size and energetic and total lipid content. These additional metrics of egg quality were intended to assist in our investigation of possible mechanisms underlying variation in embryo hatching success and also to determine whether other, more easily collected measures of egg quality could be used in future studies to accurately predict embryo hatching success.

Because larger eggs generally produce larger offspring that have higher rates of survival than small eggs^35^, we used egg size as a proxy of egg quality. To do so, we estimated the mass of an individual egg (nearest 0.01 mg) by taking the inverse of our eggs per gram of ribbon metric (see Fecundity section above). Because estimated individual egg mass is prone to possible overestimation, as it depends on ribbon mass, we also used independent measurements of individual egg diameters in a subsample of each female's egg mass that was preserved in 3% buffered glutaraldehyde immediately after spawning (*N=*20 eggs per female) as a way to check for this bias^54^. Both proxies of egg size showed similar significant differences between our short and long winter-duration treatments^54^, thus providing strong evidence that egg size differed between our treatments and was not due to differences in ribbon mass among females or treatments.

We measured the energetic content of ovaries in two ways. First, we quantified the total energetic content of egg ribbon subsamples using bomb calorimetry^66^. In brief, ovaries were dehydrated in a drying oven (65–70 °C; 48–72 h), homogenized into a fine powder, compressed into small pellets (*N=*2–3 replicates per ovary) and combusted in a Parr oxygen bomb calorimeter (Parr Instrument Company, Moline, IL). Second, we quantified the percentage of total lipids in each hand-stripped egg mass. Total lipids were extracted from ovaries after homogenization in chloroform-methanol according to refs 67, 68. The organic solvent was evaporated under a stream of nitrogen and the lipid content determined gravimetrically. To quantify energetic and lipid content on a per egg basis, we divided the total energetic and lipid content of each sample by the wet mass of the sample (expressed as calories or lipids (mg) per gram of wet mass). We then multiplied this wet mass concentration by the total ovary mass and divided by the fecundity, previously determined for each female, to convert wet mass concentrations into calories or lipids (μg) per egg, respectively.

*Larval size-at-hatching*. To determine whether larval size-at-hatching differed between winter-duration treatments, we measured three metrics of size and quality (that is, energy reserves) in preserved larvae: total length (nearest 0.1 mm); body depth at the insertion of the anal fin (nearest 0.1 mm); and yolk-sac volume (that is, a measure of energetic reserves; nearest 0.01 mm^3^). To quantify yolk-sac volume, we measured the length and width of the yolk-sac and converted these measurements into volume using the standard equation for a prorate spheroid, which closely approximates the shape of the yolk-sac in many larval fishes^69^. Measurements of preserved larvae were made using an image analysis system that consisted of a Nikon SMZ800, Digital Sight DS-U3 dissecting microscope and Nikon NIS-Elements BR v.4.00.07 software.

*Over-winter growth*. To quantify female somatic (that is, non-reproductive) growth during our winter experiment, we used data from two sources: (1) females euthanized during the first week of October 2011(the start of our winter experiment); and (2) data collected from PIT-tagged females during the course of the experiment. Females euthanized in October (*N=*9) were weighed (nearest 0.1 g wet mass) and gonads dissected to determine gonad mass (nearest 0.1 g). For these euthanized females, gonad mass ranged from 1 to 4% of total female mass (nearest 0.1 g) and was positively related to total female mass (linear regression: gonad mass=0.036 × *female mass*−0.798; *R*^2^=0.59, *P*=0.015, *N*=9). Using this relationship, we estimated the gonad mass for each female at the start of our winter experiment in early October. By then subtracting this estimated gonad mass from the total mass of each female, we obtained an estimate of each female's somatic mass at the start of the experiment. After each female had spawned (that is, lost mass associated with eggs) at the conclusion of the experiment, we subtracted the spent ovary mass from the total mass of each female to quantify somatic mass at the end of the experiment. We used the difference between somatic mass at the start and end of our winter-duration experiment as our measure of over-winter growth for female yellow perch that were successfully hand-stripped of eggs.

Using this approach, we found that all females lost somatic mass during the experiment, with the amount of mass lost (g) being negatively related to the somatic mass of females (g) measured during the first week of October 2011 (linear regression: over-winter growth=−0.25 × female mass+13.7; *R*^2^=0.58, *P*<0.001, *N*=16). To determine whether over-winter growth differed between our experimental treatments, we used residuals from this relationship as our response variable.

*Data analysis*. Before testing whether fecundity, egg quality, embryo hatching success, larval size-at-hatching and over-winter growth differed between winter-duration treatments, we tested whether each response variable was related to female size (TL), age and condition (residual mass from a log_10_(wet mass)−log_10_(TL) relationship, calculated separately for both spring [at time of spawning] and fall [when winter-duration treatments began]); previous research has shown that egg and larval traits are often correlated with female spawner size and age^70^. In addition, we tested whether embryo hatching success and larval-size-at-hatching were related to incubation temperature (calculated as the mean of mean daily temperature measured during the incubation period for each egg mass). When significant relationships were identified, we used residuals from these relationships as our response variables in our final analyses, which allowed us to remove the effect of female size, age, condition or incubation temperature (see Supplementary Table 3 for raw data).

Once the appropriate response variables had been identified and calculated, we used generalized linear mixed models (GLMMs; PROC MIXED SAS v.9.3) to test for tank effects. Tanks were included in GLMMs as random categorical effects (that is, replicates nested within winter-duration treatment, which was considered a fixed categorical effect in our models). Finding no tank effects for any of our response variables (all *P*>0.05), we calculated means of each response variable by tank (our experimental unit) and proceeded with testing for treatment effects using generalized linear models (PROC GLM, SAS v.9.3). Finally, we used regression techniques (linear and non-linear) to evaluate the relationship between embryo hatching success and our metrics of egg quality (that is, egg size, energetic and lipid content). Before conducting statistical tests (*α*=0.05), we verified that each response variable was normally distributed. We also analysed residuals from each model to verify that assumptions of normality, constant variance, independence and (when appropriate) linearity were met.

All field collections and laboratory experiments were conducted according to animal use guidelines outlined in IACUC protocol # 2009A0073 at The Ohio State University.

## Additional information

**How to cite this article:** Farmer, T. M. *et al*. Short winters threaten temperate fish populations. *Nat. Commun.* 6:7724 doi: 10.1038/ncomms8724 (2015).

## Supplementary Material

Supplementary Information Supplementary Figure 1, Supplementary Tables 1-3 and Supplementary References
