# Supplementary material for: Short winters threaten temperate fish populations
Source: Nat Commun. 2015 Jul 15;6:7724. doi: 10.1038/ncomms8724 (PMC4518244; doi:10.1038/ncomms8724)
Supplement: Supplementary Information — Supplementary Figure 1, Supplementary Tables 1-3 and Supplementary References [file ncomms8724-s1.pdf]

Supplementary Figure 1. Lake Erie juvenile yellow perch abundance versus winter water temperature prior to spawning, 1969-1992.

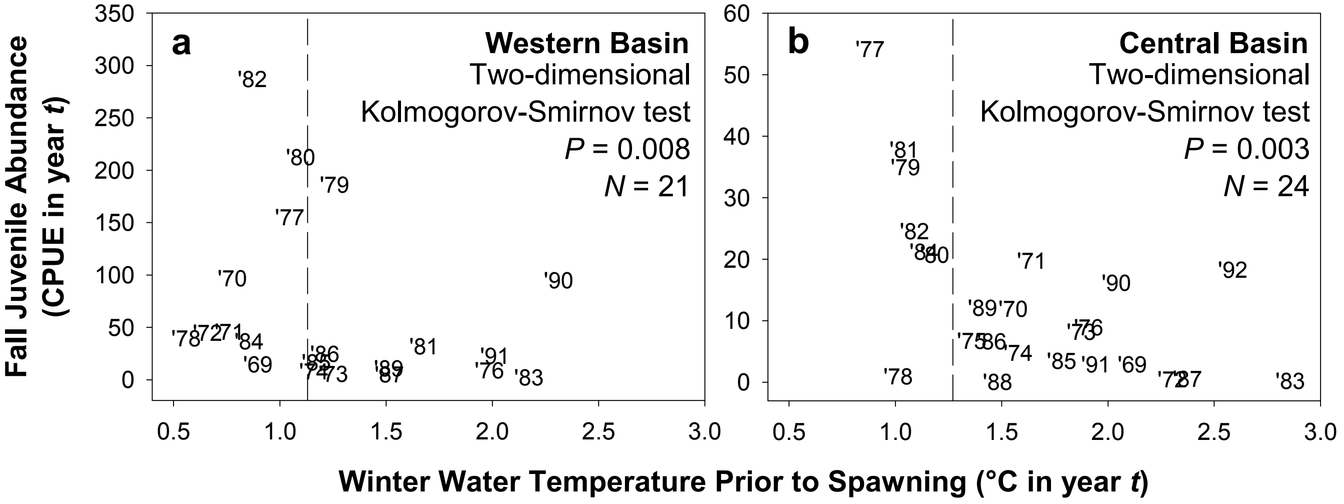

Daily winter water temperature values were obtained from historical monitoring stations in (**a**) western (Put-in-Bay, OH) and (**b**) central (Erie, PA) Lake Erie[1] and averaged for winter months (January-March). In (**a**) western Lake Erie, large data gaps existed during the winters of 1975, 1988, and 1982, preventing these years from being used in the analysis. Two-dimensional Kolmogorov-Smirnov tests[2] determined if changes in the variance of juvenile abundance in (**a**) western and (**b**) central Lake Erie were related to water temperature from the previous winter. This distribution-free test identifies a threshold value of ice cover (indicated by vertical dashed lines) that maximizes the difference in variance of juvenile abundance between the two sides of the threshold. *P*-values indicate if the variance of juvenile abundance differs significantly between the two sides of the threshold. Below the threshold, juvenile abundance may be high or low, but above the threshold only low abundances occur. Thus, winter duration appears to 'set the stage' for future high recruitment to the fishery. Juvenile abundance was determined from annual, fisheries-independent Ohio Department of Natural Resources-Division of Wildlife bottom trawling surveys conducted during October of year *t*, and is presented as catch-per-unit-effort (CPUE; # of individuals per trawl min) for both (**a**) western and (**b**) central Lake Erie.

Supplementary Table 1. Additional results from a controlled laboratory experiment testing the effect of winter duration on Lake Erie yellow perch reproductive success.

Response Variable	Female Size	Female Age	Spring Female Condition	Fall Female Condition	Incubation Temperature
Fecundity	**<.0001** (17)	0.99 (16)	0.29 (17)	0.28 (16)	-
Egg quality					
Individual egg mass (mg)*	0.24 (17)	0.10 (16)	0.24 (17)	0.15 (16)	-
Energetic content (cal) per egg	0.19 (17)	0.23 (16)	0.38 (17)	0.71 (16)	-
Total lipid content (μg) per egg	0.47 (14)	0.09 (13)	0.27 (14)	0.99 (13)	-
Embryo hatching success (%)	0.67 (17)	0.23 (16)	0.24 (17)	0.55 (16)	0.60 (17)
Larval size-at-hatching					
Larval total length (mm)	0.55 (17)	0.88 (16)	0.78 (17)	0.99 (16)	0.92 (17)
Larval body depth (mm)	0.66 (17)	0.48 (16)	0.76 (17)	0.54 (16)	0.89 (17)
Larval yolk sac volume (mm^3)	0.14 (17)	0.29 (16)	0.87 (17)	0.78 (16)	0.71 (17)

Associated *P*-values for Pearson correlation coefficients testing the response of fecundity (total eggs per female), egg mass, egg composition (i.e., energetic and total lipid content per egg), embryo hatching success, and larval size-at-hatching to indices of female yellow perch size (nearest 1 mm total length, TL), age, and condition (both spring and fall separately) and incubation temperature (ºC). Female condition in spring and fall was calculated as a residual of a $\log_{10}$(total mass)-$\log_{10}$(total length) relationship derived separately in both the spring (time of spawning) and fall (initiation of winter experiment). In both the spring and fall, only female somatic mass (total body mass – egg mass) was used in this relationship. Bold values indicate $P < 0.05$. Values in parentheses represent sample sizes. These results show that all of our metrics of egg and larval quality were unrelated to individual female attributes and incubation temperatures. *****Responses significantly affected by winter-duration treatments had data adjusted for treatment effects prior to estimating correlations.

Supplementary Table 2. Parameter estimates and associated Wald χ^2 and *P*-values for logistic regressions.

**Year**	***N***	**Parameter**	**Estimate**	**Wald χ^2**	***P***
2010	398	β_0	-9.29	54.07	< 0.0001
		β_1	0.85	47.53	< 0.0001
2011	348	β_0	-8.44	88.33	< 0.0001
		β_1	0.80	77.53	< 0.0001
2012	210	β_0	-49.59	17.57	< 0.0001
		β_1	4.73	16.76	< 0.0001

Logistic regressions related the probability that a mature Lake Erie yellow perch was spent to spring (April-May) bottom water temperature during 2010-2012. The logistic regression model used was $\text{logit}(\pi_i) = \beta_0 + \beta_1 X_i$ where β_0 was the intercept, B_1 was the slope, and X_i was bottom water temperature. *N* is the number of mature female yellow perch used in each analysis.

Supplementary Table 3. Raw data used to assess correlations among predictor and response variables from a controlled laboratory experiment testing the effect of winter duration on Lake Erie yellow perch reproductive success.

ID	Winter Duration	Total Length (mm)	Age	Spring Female Condition	Fall Female Condition	Incubation Temperature (°C)	Fecundity (total eggs)	Individual Egg Mass (mg)*
1	Short	258	5	0.005	0.010	14.6	37,992	2.05
2	Short	222	5	-0.007	-0.017	14.7	12,730	4.15
3	Short	286	6	0.014	-0.035	15.0	44,233	3.26
4	Short	226	5	0.017	0.051	13.5	27,894	2.59
5	Short	224	.	-0.021	0.017	15.4	14,731	3.81
6	Short	267	6	0.009	-0.025	15.9	45,151	2.75
7	Short	237	3	-0.002	0.003	16.9	28,249	2.75
8	Long	225	4	-0.014	0.009	16.5	24,570	2.85
9	Long	253	5	0.006	0.048	16.6	27,464	3.66
10	Long	251	5	0.008	0.021	16.6	27,239	3.00
11	Long	226	5	0.010	-0.010	17.3	16,965	3.26
12	Long	273	5	0.006	0.030	17.2	39,596	2.72
13	Long	230	5	0.006	-0.001	17.3	17,678	3.72
14	Long	227	6	0.005	.	17.5	22,579	2.91
15	Long	262	5	-0.005	0.085	17.7	54,441	2.19
16	Long	277	7	-0.011	0.031	17.6	35,233	3.04
17	Long	250	9	-0.007	0.003	17.1	20,084	4.24

Predictor variables include: female size, age, condition, and incubation temperature. Response variables include: fecundity, egg size, egg energetic and lipid content, embryo hatching success, and larval size-at-hatching. Total length was measured at time of spawning for each female. Female condition in spring and fall was calculated as a residual of a $\log_{10}$(mass)-$\log_{10}$(total length) relationship derived separately in both the spring (time of spawning) and fall (initiation of winter experiment). In both the spring and fall, only female somatic mass (total body mass – egg mass) was used in this relationship. *Values for individual egg mass were adjusted to account for the significant effect of winter duration on this response variable.

Supplementary Table 3 (Continued). Raw data used to assess correlations among predictor and response variables from a controlled laboratory experiment testing the effect of winter duration on Lake Erie yellow perch reproductive success.

ID	Embryo Hatching Success (%)	Energetic Content (cal) Per Egg	Total Lipid Content (μg) Per Egg	Larval Total Length (mm)	Larval Body Depth (mm)	Larval Yolk Sac Volume (mm^3)
1	1%	1.31	.	4.81	0.32	0.68
2	84%	3.10	.	5.47	0.36	0.95
3	78%	2.14	0.11	5.30	0.36	0.64
4	12%	1.78	0.09	5.20	0.34	0.77
5	80%	2.76	0.14	5.23	0.37	0.77
6	3%	1.82	0.09	4.38	0.31	0.70
7	1%	1.74	0.08	4.69	0.29	0.67
8	94%	2.42	0.13	5.32	0.36	0.63
9	78%	3.25	0.14	5.19	0.35	0.86
10	55%	2.80	0.14	5.13	0.35	0.89
11	62%	3.04	0.13	5.30	0.36	0.68
12	91%	2.11	.	5.72	0.37	0.63
13	15%	3.38	0.14	5.37	0.37	0.82
14	77%	2.32	0.13	5.11	0.33	0.86
15	14%	2.15	0.11	4.78	0.30	0.61
16	36%	2.33	0.12	5.25	0.36	0.78
17	100%	3.42	0.17	5.12	0.34	0.83

Predictor variables include: female size, age, condition, and incubation temperature. Response variables include: fecundity, egg size, egg energetic and lipid content, embryo hatching success, and larval size-at-hatching.

Supplementary References

1. McCormick, M. J. & Fahnenstiel, G. L. Recent climatic trends in nearshore water temperatures in the St. Lawrence Great Lakes. *Limnol. Oceanogr.* **44**, 530-540 (1999).

2. Garvey, J. E., Marschall, E. A. & Wright, R. A. From star charts to stoneflies: Detecting relationships in continuous bivariate data. *Ecology* **79**, 442-447 (1998).
